# Geotechnical and Environmental Assessment of Blast Furnace Slag for Engineering Applications

**DOI:** 10.3390/ma14206029

**Published:** 2021-10-13

**Authors:** Wojciech Sas, Justyna Dzięcioł, Algirdas Radzevičius, Maja Radziemska, Midona Dapkienė, Raimondas Šadzevičius, Rytis Skominas, Andrzej Głuchowski

**Affiliations:** 1 SGGW Water Centre, Warsaw University of Life Sciences-SGGW, 02787 Warsaw, Poland; wojciech_sas@sggw.edu.pl; 2 Institute of Civil Engineering, Warsaw University of Life Sciences-SGGW, 02787 Warsaw, Poland; 3 Institute of Hydraulic Engineering, Vytautas Magnus University Agriculture Academy, 53361 Kaunas, Lithuania; algirdas.radzevicius@vdu.lt (A.R.); midona.dapkiene@vdu.lt (M.D.); raimondas.sadzevicius@vdu.lt (R.Š.); rytis.skominas@vdu.lt (R.S.); 4 Institute of Environmental Engineering, Warsaw University of Life Sciences, Nowoursynowska 159, 02776 Warsaw, Poland; maja_radziemska@sggw.edu.pl

**Keywords:** oedometric, anthropogenic, aggregate, heavy metals, blast furnace slag, compression

## Abstract

The increasing demand for building materials in the road industry creates interest for a new source of high-quality aggregates. In order to conserve natural resources, more attention is focused on anthropogenic soils and industrial solid wastes. For the successful application of these types of soil, a series of geotechnical and environmental tests have to be conducted. A potential hazard in the reuse of wastes from thermal degradation in the construction industry, particularly in reinforced concrete (RC) construction, is the migration of heavy metals into the groundwater environment. In this article, a geotechnical assessment of blast furnace slag (BFS) properties is presented. We conducted a series of CBR, and oedometric tests to evaluate the feasibility of BFS application in earth construction. The oedometric test results show acceptable compression characteristics which are in the range of natural aggregates. The CBR shows that this material may be used as a pavement subbase. We also noticed the preconsolidation pressure phenomenon in both Proctor and vibro-compacted soil during the oedometric test. The compression index and recompression index value show that the compression characteristics are close to those of dense sand. Based on the results described in the article, blast furnace slag is a candidate for technological application and can become one of the elements of sustainable development by contributing to a reduction in the negative environmental impact of production and use of building materials.

## 1. Introduction

The dynamic development of construction infrastructure in the world in recent decades has resulted in a high demand for unbound aggregates. A decrease in the resources of natural aggregates has necessitated the search for their substitutes and alternatives [1,2].

Anthropogenic materials (AMs) used in construction can be divided into several categories according to the origin of the material [3]. One category of aggregates that fits into the definition of anthropogenic soil is industrial solid waste (ISW), which is a group of materials derived from industrial byproducts. Examples of such products are magnesium slag, fly ash, blast furnace slag, or steelmaking slag. The detailed classification, characteristics, and properties of ISW can be found in [4]. Blast furnace slag (BFS) is a particularly interesting anthropogenic material. This material is a byproduct of steel mills, which is deposited in slag dumps at the end of the process [5,6]. The slag cooling technique impacts the type of generated slag, which leads to further dividing of the BFS. The air-only-cooled BFS produces crystallin slag, the BFS cooled by large quantities of water or air results in ground-granulated BFS (GGBFS), and the BFS cooled by controlled quantities of water or steam produces expanded slag [7].

The beginning of the use of ground slag in the construction industry can be traced back to the early 20th century. At that time, ground slag was used as a cementitious material in concrete. Ground-granulated blast furnace slag (GGBFS) is also used in composite cements. In the late 1950s, GGBFS began to be used as a separately ground material added to the concrete mixer along with Portland cement [8]. Currently, the application of GGBFS as a binder, as an industrial byproduct, is well established and finds applications as an additive or replacement for cement. Its effects on durability have been confirmed, including resistance to sulfate attack or high resistance to chloride penetration [9,10]. BFS has latent hydraulic properties which allows its use as a cement additive [11]. The engineering properties of BFS vary and are mostly determined by the cooling rate of molten slag. BFS is glassy, sand-like material [12]. However, some applications of BFS as engineering materials have proven to be unsuccessful, which has lowered the level of confidence in this material [13,14].

In recent years, there has been a significant increase in awareness and emphasis on protecting the environment and its resources. This is due to the substantial degradation of the environment due to human industrial activities. This has also had an impact on the tightening of European Union regulations on waste disposal and has stimulated the search for new ways of recycling [15,16,17,18].

Blast furnace slag and combustion waste landfills are generally regarded as an environmental nuisance. Their main negative environmental impacts include the impact of infiltrating water from the landfill on groundwater, alteration of surface water quality by discharge of infiltrating water from the landfill, and pollution of air and adjacent land and plants by dust carried from the surface of the landfill [19,20,21].

Considering the above issues, it is necessary to analyze the suitability of GGBFS in earth construction, in terms of both its mechanical properties and its environmental impact. Due to the wide variety and non-standardization of anthropogenic materials including GGBFS, it is necessary to continuously increase the knowledge about the impact and properties of these materials.

GGBFS application in pavement engineering and earth embankment construction needs preliminary tests to evaluate compaction potential and bearing capacity properties. The bearing capacity for pavement engineering purposes is tested using the California Bearing Ratio (CBR) test. Previous studies have reported that GGBFS has a sufficiently high CBR value for its use as a pavement subbase [7,22].

The application of 3–12% GGBFS in a mix with soft soil has shown a significant impact on the physical and mechanical stabilized soil properties. BFS addition increases the maximum dry density (MDD) and decreases the optimal moisture content (OMC). Furthermore, BFS addition decreases the swelling behavior, increases the CBR from 8–18% for unsoaked samples and from 2–10% for soaked samples, and improves the unconfined compressive strength (UCS) from 118 kPa to 153 kPa [23].

BFS has been proven as a reliable soil stabilization material with a particular focus on expansive soils. GGBFS addition alters the soil grain size distribution, which results in a decrease in clay fraction share and lowers the plasticity limit. Moreover, the soil specific gravity increases and a reduction in swell potential is observed [24].

BFS is also used as a stabilization mix material. The studies of a soil mix consisting of BFS and slag/fly ash/burn clay pozzolana revealed that alluvial soil stabilized with such a mix (20% of mass content) increased its strength by three times when compared to natural soil, with 7.5% gypsum content in the soil mix [25]. The non-cohesive soil stabilization of desert silty sands with BFS (5% to 15%) and lime (1% to 5%) addition led to a significant improvement in soil mechanical properties. The CBR value increased from 33.2% for untreated compacted soil to 175% in unsoaked and 140% in soaked conditions for an optimal mix of 15% BFS and 5% lime content. The UCS for optimal mixture reached 4.0 MPa after 28 days of curing time [26].

The stabilization technique is frequently used mostly due to the presence of water; furthermore, lime and cement stabilization with the addition of fly ash or BFS is beneficial in lowering the water content, which contributes significantly to the compaction effort [27,28,29,30,31,32].

The internal friction angle of BFS is 39°–43°, and the specific gravity ranges between 2.29 and 3.35 [12,33,34]. The results of CBR tests on GGBFS revealed that the bearing capacity affects the soil arrangement and interparticle forces such as matric suction and dilatancy. The CBR ranges between 65% to 160% for the standard and modified Proctor compaction techniques [34].

GGBFS is mainly composed of silicates and aluminosilicates of calcium melt that were historically removed from the blast furnace. The chemical composition of GGBFS depends on the materials used to produce the iron, while the physical properties depend on the cooling process used to cool the molten materials. During the cooling process, amorphous glassy granules of various sizes are formed by quenching in water using a high-pressure water jet [35].

The chemical composition of GGBFS depends on the composition of the raw materials. It mainly contains silica (SiO_2_) 33–36.6%, alumina (Al_2_O_3_) 12–14.2%, and calcium (CaO) 32–43.9%, which account for 77–94.5% of the composition [35,36,37]. The main chemical components of GGBFS are similar to Portland cement.

Some of the geotechnical problems require an evaluation of not only the strength properties but also the compression characteristics. The compression of anthropogenic materials is an important problem that has to be addressed with the same attention as the topic of soil strength.

In this article, the results of a geotechnical and geoenvironmental program with the aim of characterizing GGBFS for engineering applications are presented.

## 2. Materials and Methods

### 2.1. Soil Gradation

**Figure 1:**
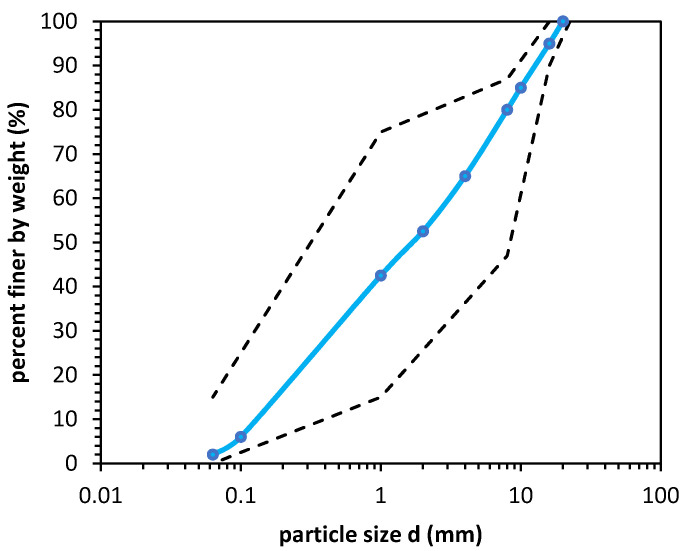
Soil gradation curve for ISW tested in this study (solid blue line); the black dashed lines represent the Polish standard WT-4 requirements for subbase soils.

GGBFS in this research was obtained from the Lafarge Cement SA cement plant, (Warsaw, Poland) created in the blast furnace process. The anthropogenic soil was first sieved into the appropriate fractions (according to ISO 17892-4:2016), and then a soil gradation curve was composed from sieved material. The composed soil gradation curve fit most pavement design codes. The soil gradation curve had a 47.5% gravel fraction, 50.5% sand fraction content, and 2% fine content. The coefficient of uniformity C_U_ was equal to 30, and the coefficient of curvature C_C_ was equal to 0.53. The composed soil was well graded and was recognized as gravelly sand (grSa) according to EUROCOD 7 (PN-EN 1997-2:2009, PN-EN ISO 14688-2:2006). The soil gradation curve is presented in Figure 1.

### 2.2. Compaction and CBR Tests

Our laboratory CBR tests were conducted using two different compaction methods, namely, the Proctor method and the vibro method. The Proctor method was conducted according to the American Society for Testing and Materials (ASTM) standard (ASTM D698-12e1). The Proctor method is characterized by the use of a 2.5 kg hammer and a larger mold of 150 mm diameter and 120 mm height, with a volume of 2.2 dm^3^. A three-layer Proctor test was performed, with 56 blows to each layer. The vibratory compaction test (vibro method) was conducted using a vibratory compaction hammer. The compaction was conducted in three layers, with 8 s excitation on each layer to ensure that the energy of compaction corresponded with the Proctors test energy of compaction (energy of compaction equal to 0.59 J/cm^3^). The results of the tests led to the determination of maximum dry density (MDD) and optimum moisture content (OMC). The CBR test was conducted with a standard penetration rate of 1.25 mm/min, and the penetration was recorded up to 2.54 mm.

### 2.3. Oedometer Tests

The oedometer consolidation test was adopted in this study for the determination of the compressibility of the tested materials when subjected to vertical loads. The results were used to calculate and estimate the oedometric modulus *E_oed_* and preconsolidation pressure *p*’*_c_*. Those parameters are used for settlement calculations. In this test, compacted soil specimens loaded vertically in constant stress steps were tested to characterize the primary consolidation.

The oedometric tests of specimens took place in the Proctor cylinder (d = 150 mm, h = 120 mm), which supported no lateral movements of the soil. The following sequence of loading steps was applied: 12.5, 25, 50, 100, 200, 400, 800, and 1600 kPa. Each increment in loading was held constant for 100 s. After the first loading, the unloading process was done in one step to a pressure of 50 kPa. Next, the reloading process was initiated at the value of 1600 kPa, with the first loading up to 3200 kPa.

### 2.4. Geoenvironmental Tests

The environmental impact of the GGBFS material was evaluated by testing the chemical composition and heavy-metal content. The study was performed by atomic absorption spectroscopy (AAS), the measurement of mercury content was performed using an atomic absorption spectrometer with the amalgamation technique using an AMA 254 mercury analyzer.

## 3. Results

### 3.1. Geoenvironmental Tests Results

**Figure 2:**
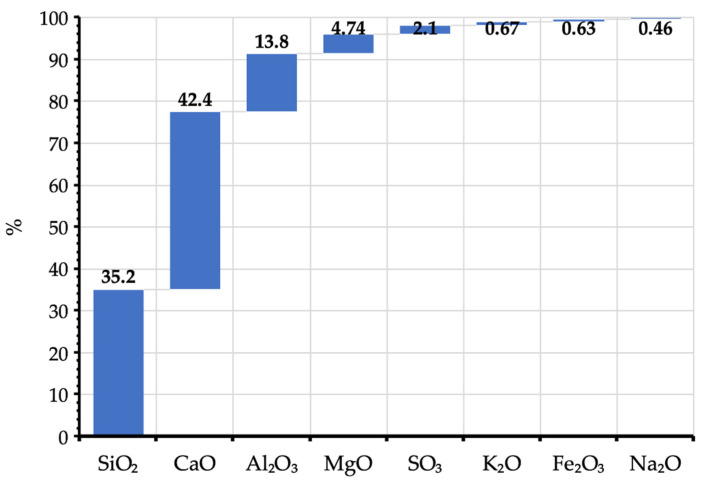
Results of the chemical composition test for GGBFS.

The composition of ground-granulated blast furnace slag is variable and may depend, for example, on its cooling method. The main mineral compounds contained in the tested material were silicon dioxide (SiO_2_) and calcium oxide (CaO). Together, they accounted for 77.6% of the total composition. In addition, a significant alumina (Al_2_O_3_) content of ~14% was identified. The chemical composition of the material is presented in Figure 2.

Due to the potential use of GGBFS in exposure to soil–water environments, it was tested for heavy-metal content. The results are presented in Table 1.

According to the Regulation of the European Parliament and of the Council No 1272/2008 of 16 December 2008 [38], it was estimated that the contents of zinc, lead, and copper were not exceeded, indicating a low impact of those metals on the environment if this material were to be used as the basic substructure.

Some doubts may be raised by the increased contents of cadmium and mercury. Cadmium is characterized by high mobility in acidic environments and accumulates mainly in the roots and green parts of plants [39,40]. Environmental mercury pollution is produced by anthropogenic and natural factors, causing its bioaccumulation. Mercury changes its chemical form in the environment and moves from place to place, eventually depositing itself deep in soils and sediments [41].

Considering the applied characteristics of GGBFS and its high pH value of 11.49, the mobility of these metals into the environment would be inhibited. This is due to the properties of mercury and cadmium, whose geochemical activity decreases with increasing pH. The mobility and activity of cadmium and mercury decrease at pH = 4.5–6 [39,40,41,42]. Therefore, it can be concluded that their content would not preclude the possibility of using this material in road construction.

### 3.2. Proctor Test Results

The results of the compaction tests show that OMC was equal to 10.7% for the vibro compaction method and 10.2% for the Proctor compaction method.

**Figure 3:**
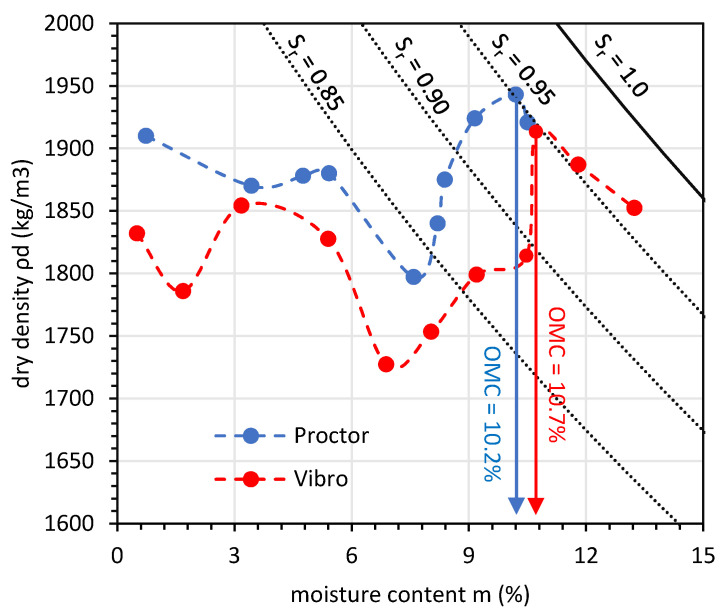
Results of the compaction tests for GGBFS.

The MDD was equal to 1.91 g/cm^3^ for vibro compaction and 1.95 g/cm^3^ for the Proctor method. The specific gravity for GGBFS was equal to 2.91. Figure 3 presents the compaction curves for GGBFS using the two compaction methods.

The compaction curve indicates that there existed one point at which OMC could be determined, although, as for non-cohesive soil, on the dry side of the compaction curve, a high dry density could be observed. The results of the compaction test for GGBFS indicate a higher OMC than for natural aggregates with the same soil gradation characteristics [43]. This might have been a result of higher surface grain roughness. It is worth noting that, in both cases, after reaching a certain point of moisture content, the dry density increased rapidly. With the rising moisture content, the water coat on the grain surface became thick enough to overcome the surface friction and water suction (note that the saturation ratio was over 0.85 in both cases). The Proctor compaction exhibited this phenomenon at lower moisture content. The Proctor test is an impact test, which means that the energy transmitted with one blow could be high enough to overcome the pore water suction, whereby the phenomenon would occur at a lower moisture content than in the case of vibro compaction.

### 3.3. CBR Test Results

**Figure 4:**
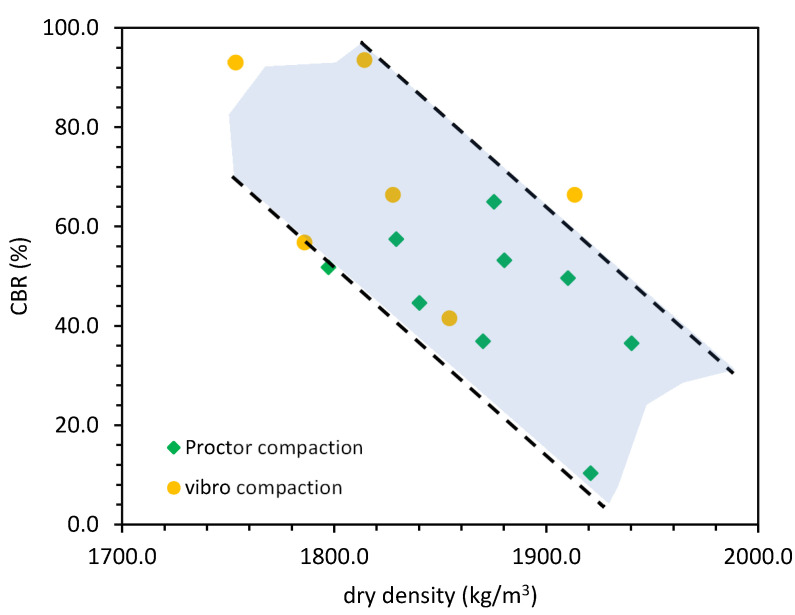
The CBR–dry density relationship of GGBFS in this study.

The CBR tests were carried out to evaluate the bearing capacity for pavement engineering application of GGBFS. The results of CBR show that, from the bearing capacity point of view, GGBFS may be applicated as a subbase [44]. Nevertheless, the results show that this type of soil showed an inverse CBR–dry density relationship. The highest CBR equal to 93.1% was in the region of lowest dry density from the vibro compaction tests (see Figure 4).

Such characteristics might have been caused by the crushability of GGBFS, which would be higher in higher dry density states.

**Figure 5:**
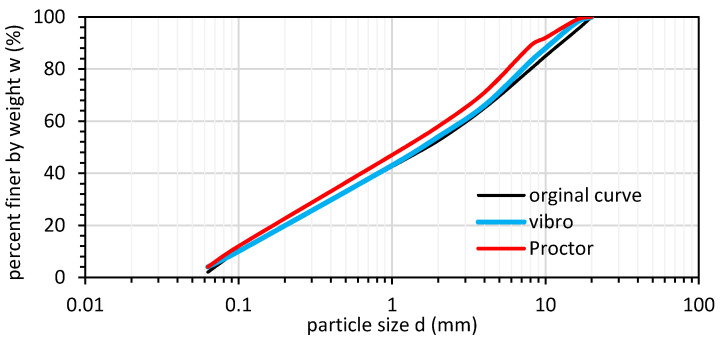
The crushability test results for GGBFS after Proctor and vibro compaction at an optimal moisture content.

The vibro-compacted samples had a higher bearing capacity than the Proctor-compacted samples according to the CBR bearing capacity tests. The CBR value from vibro-compacted samples was 1.47 times higher than the highest CBR from the tested Proctor-compacted samples. To check if the GGBFS was susceptible to crushing, we checked the fraction share change after compaction in the CBR mold for the Proctor and vibro compaction tests (see Figure 5). The laboratory results are presented, and additional field tests are required to confirm this phenomenon.

The test results show that GGBFS was crushed during compaction, especially during Proctor compaction. The compaction tests were performed at an optimal moisture content. The fraction share change indicates that the GGBFS fraction which was crushed the most was the coarse fraction (20–10 mm). Since the Proctor method uses an impact test during compaction, the coarse grains were more prone to crushing. At a high moisture content, the GGBFS was less resistant to compaction; thus, during the impact, the coarse grains absorbed most of the blow. Furthermore, the higher CBR value might have been responsible for the higher friction between grains and particles and the water suction effect at a low moisture content.

The abovementioned phenomena lead to the conclusion that a high moisture content was less beneficial to the GGBFS CBR bearing capacity. It is worth noting that the CBR tests were conducted right after compaction, which leads to the question of what the CBR value would have been if the GGBFS sample had been left to dry.

### 3.4. Oedometric Test Results

**Figure 6:**
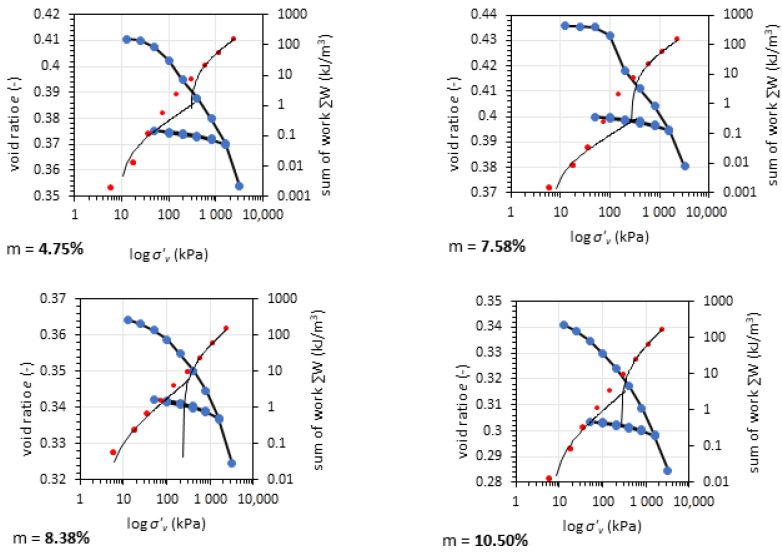
Results of oedometric tests for GGBFS at different moisture content compacted using the Proctor normal energy effort. Red dots represent the work method for calculation of preconsolidation pressure; blue dots represent the oedometric test results.

The oedometric tests give information about the soil compressibility. Often, in pavement engineering, most of the information about the soil concerns the bearing capacity with little or no reference to the soil settlement or the soil deformation potential. Therefore, to fully characterize this material, oedometric tests are presented for GGBFS in various compaction and moisture conditions. The tests were conducted in unsoaked conditions. Figure 6 presents the results of the oedometric test for GGBFS after Proctor compaction.

**Figure 7:**
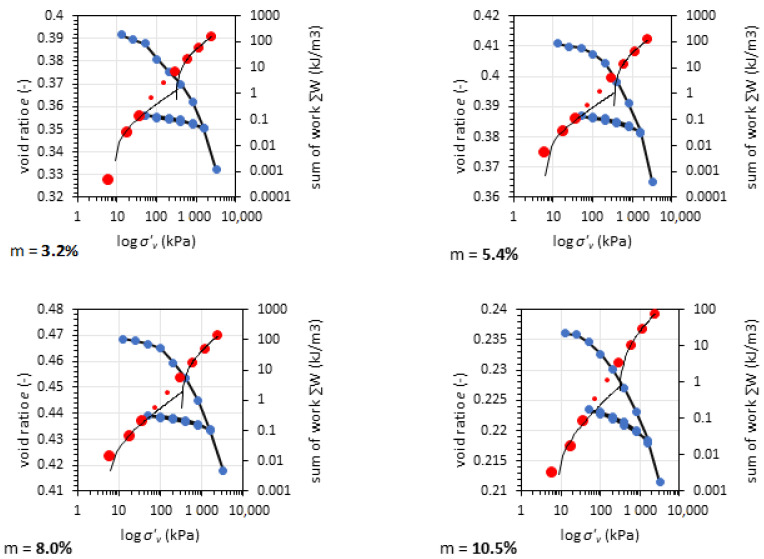
Results of oedometric tests for GGBFS at different moisture content compacted using the vibro method with normal energy effort. Red dots represent the work method for calculation of preconsolidation pressure; blue dots represent the oedometric test results.

The GGBFS preconsolidation pressure *p*’*_c_* was in the range from 277.9 to 360.6 kPa according to the calculations [43]. A higher preconsolidation pressure (about 20–30 kPa) in vibro-compacted samples was observed. The tangent oedometric modulus value *E_ode_* for the vertical stress *σ*’*_v_* between 800 and 1600 kPa was between 95.2 and 134.2 MPa. The detailed results of the oedometric tests for GGBFS are presented in Table 2. Figure 7 shows the results of oedometric tests for GGBFA compacted using the vibratory method.

**Figure 8:**
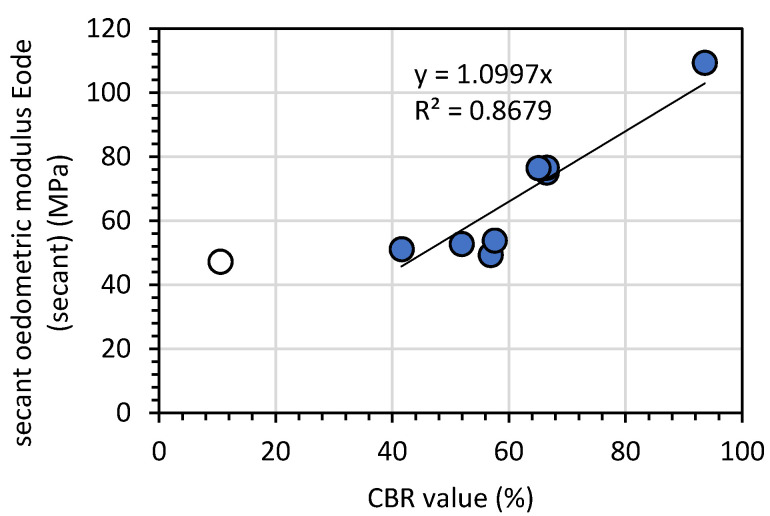
The relationship between the secant oedometric modulus *E_ode (secant)_* and the CBR value for GGBFS. The empty point denotes a test excluded from the analysis.

The preconsolidation pressure for both types of compaction methods shows that GGBFS was preconsolidated. The oedometric modulus *E_ode_* for Proctor compaction was between 277.9 and 315.6 MPa, while that for Vibro compaction was between 316.0 and 360.6 MPa The *p*’*_c_* was higher for the vibro method, which may have been caused by horizontal movements of grains during the compaction process. The *E_oed (tangent)_* values were calculated for first loading in the range of 800 to 1600 kPa. For Proctor-compacted samples, *E_oed (tangent)_* values were more consistent but lower than the maximal *E_oed (tangent)_* value for vibro compaction. The secant oedometric modulus *E_oed (secant)_* revealed soil compressibility in the range of 12.5 to 1600 kPa. Due to the fact that the soil loading in the CBR test and oedometric test was restrained to horizontal displacement, we compared the CBR and *E_oed (secant)_* values to establish their relationship (see Figure 8).

The CBR value corelated with the *E_oed (secant)_* value, and their relationship can be described as a linear function where *E_oed (secant)_* = 1.1·CBR, which is similar to the specific Green and Hall formula in power form E = a·CBR^b^, where b = 1 [45,46].

**Figure 9:**
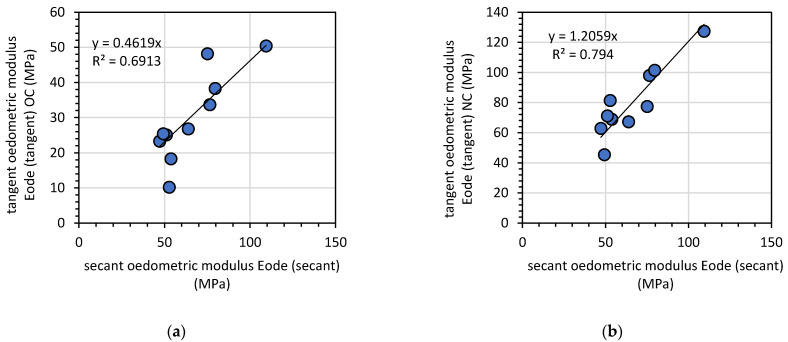
The relationship between the secant oedometric modulus *E_ode (secant)_* and the tangent modulus below (**a**) and above (**b**) the preconsolidation pressure *p*’*_c_*.

The tangent oedometric modulus was divided to two zones limited by the preconsolidation pressure *p*’*_c_*. The modulus for a stress level below *p*’*_c_* was denoted as *E_ode (tangent)OC_*, and the stress over the preconsolidation limit was denoted as *E_ode (tangent)NC_*. The analysis of the relationship between the tangent and secant modulus led to the establishment of a relationship between the modulus values presented in Figure 9.

The tangent modulus for stress levels above the preconsolidation pressure was higher than the secant modulus; in this case, it was around 1.21 times higher than *E_ode (tangent) NC_*, whereas *E_ode (tangent) NC_* was 0.46 times lower than the secant modulus.

## 4. Discussion

According to the literature review by Buddhdev and Timani [14], geotechnical surveys on GGBFS properties are limited. Most tests were conducted to study how the addition of GGBFS improves the geotechnical properties of expansive soils. The CBR of GGBFS in this study was over 60% with the highest value equal to 93.1%, which corresponds to the CBR test results conducted on a GBFS–aggregate mix [22], where CBR was between 74.2% and 104.5%, as well as to test results on a blended calcium sulfate–GGBFS stabilization mix, where the CBR value was equal to 100% 6 days after stabilization.

Environmental considerations need to take into account the long-term impact of GGBFS on the groundwater. Observations from in situ slag weathering and laboratory leaching experiments have shown that metals and metalloids can be released into the environment [47,48]. Road construction usually prevents extensive leaching from occurring in the subbase course, and the negative impact of metal release into the environment becomes less extensive. Environmental considerations need to take into account the application of GGBFS. For example, the application of GGBFS as backfill material in underground and open-pit mines would have a less damaging impact on the environment [49]. In our study, GGBFS fulfilled the environmental safety standards of the Regulation of the European Parliament and of the Council No 1272/2008.

The compaction properties of GGBFS have rarely been investigated, and studies have mostly concerned GGBFS–soil mixes that do not reflect this material’s compaction properties [11,14,50]. Our studies show that, in laboratory tests, vibro compaction gave better results in terms of bearing capacity, in agreement with tests conducted on natural aggregates [51]. We also found that crushing occurred when impact compaction was conducted, which may limit the geotechnical application of GGBFS; nevertheless, additional in situ tests are required to check if this observation is also accurate for field conditions.

The oedometric tests conducted on GGBFS showed that this material’s oedometric secant modulus is in the range of 47.2 to 109.4 MPa. The tangent modulus value is in the range of 10.2 to 127.4 MPa. For GGBFS, the tangent-constrained modulus for vertical stress equal to 200 kPa was equal to 9.5 MPa [52].

According to the tests conducted in this article, GGBFS can be used as a material in geotechnical engineering applications. The next step is to conduct in situ tests and leaching tests to confirm the presented findings.

## 5. Conclusions

The following conclusions can be drawn on the basis of the literature review and the research performed:

1. GGBFS can be used in road construction as a basic subgrade; a CBR value higher than 60% was achieved in both Proctor and vibro compaction. The results show that this type of soil shows an inverse CBR–dry density relationship. This phenomenon might be caused by the higher grain crushability at a higher compaction state.
2. The material conforms to environmental safety standards. Considering the values of cadmium and mercury content, it is recommended for use in soils with a pH above 5, which will reduce the mobility of heavy metals.
3. GGBFS has similar compaction properties to natural aggregates. The compaction curve showed that a high dry density is observed in the dry and wet states. Proctor compaction showed a higher dry density as a function of moisture content, and the OMC from the Proctor test was higher than that from vibro compaction. The compaction tests revealed a moisture content level at which the dry density increased rapidly. This phenomenon is linked to grain surface roughness. Therefore, to achieve maximal dry density, we recommend compacting the GGBFS at a high moisture content, especially because the drop in dry density on the wet side is much gentler than on the dry side.
4. The oedometric test showed that the GGBFS has a preconsolidation pressure as a result of the compaction effort. The value of preconsolidation pressure ranges between 277.9 and 360.6 kPa and depends on the compaction method. During the oedometric test in the range of applied pressure from 1600 to 3200 kPa, a crushing sound was heard. Therefore, an additional freeze–thaw test should be run on the soil to confirm the frost resistance of GGBFS.
5. The compression index value of GGBFS is in the same range as for natural dense and loose sand. The recompression index value is generally 13–20% of the Cc index, which is the same range as for natural aggregates. The oedometric modulus *E_ode_* value is close to that of sandy gravel soils.
6. The secant oedometric modulus *E_oed (secant)_* corelates with the CBR value. Both tests are performed in similar conditions; hence, the soil compression characteristics can be calculated as a linear relationship to the CBR value, which is comparable to its relationship with natural aggregates.

## Figures and Tables

**Table 1:** The results of AAS and AMA testing for heavy metals in GGBFS.

mg/kg	Zn	Pb	Cu	Cd	Hg	Ni
GGBFS	66.3	12.6	5.5	6.2	9.6	-

**Table 2:** Results of the oedometric test calculations of oedometric modulus characteristics.

	Moisture *m* (%)	*E_ode (tangent) NC_* (MPa)	*E_ode (tangent) OC_* (MPa)	*E_ode (secant)_* (MPa)	*e*_0_ (-)
Proctor	4.75	68.9	18.3	53.8	0.464
	7.58	81.4	10.2	52.8	0.490
	8.38	98.0	33.7	76.5	0.428
	10.5	62.9	23.3	47.2	0.394
Vibro	3.2	71.2	25.1	51.1	0.443
	5.4	77.4	48.2	75.11	0.465
	8.0	67.3	26.8	63.9	0.527
	10.5	127.4	50.4	109.4	0.283

## Data Availability

Data are contained within the article.
